# Association between the G/G genotype of the lncRNA *MEG3* rs7158663 polymorphism and proliferative diabetic retinopathy

**DOI:** 10.20945/2359-4292-2024-0024

**Published:** 2024-10-21

**Authors:** Leticia de Almeida Brondani, Isabele Dandolini, Eliandra Girardi, Luís Henrique Canani, Daisy Crispim, Cristine Dieter

**Affiliations:** 1 Unidade de Pesquisa Laboratorial Centro de Pesquisa Experimental Hospital de Clínicas de Porto Alegre Porto Alegre RS Brasil Unidade de Pesquisa Laboratorial, Centro de Pesquisa Experimental, Hospital de Clínicas de Porto Alegre, Porto Alegre, RS, Brasil; 2 Hospital de Clínicas de Porto Alegre Serviço de Endocrinologia Porto Alegre RS Brasil Serviço de Endocrinologia, Hospital de Clínicas de Porto Alegre, Porto Alegre, RS, Brasil; 3 Universidade Federal do Rio Grande do Sul Faculdade de Medicina Porto Alegre RS Brasil Programa de Pós-graduação em Ciências Médicas: Endocrinologia, Faculdade de Medicina, Universidade Federal do Rio Grande do Sul, Porto Alegre, RS, Brasil; 4 Universidade do Vale do Rio dos Sinos São Leopoldo RS Brasil Universidade do Vale do Rio dos Sinos, São Leopoldo, RS, Brasil

**Keywords:** Diabetic retinopathy, polymorphisms, lncRNA *MEG3*

## Abstract

**Objective:**

To investigate the association between the long noncoding RNAs (lncRNAs) *maternally expressed gene 3* (*MEG3*) rs7158663 polymorphism and diabetic retinopathy (DR) in patients with type 2 diabetes mellitus (T2DM).

**Subjects and methods:**

The study included 628 patients with T2DM and DR ("case group," including 283 with proliferative DR [PDR] and 345 with nonproliferative DR [NPDR]), and 381 patients with T2DM but no DR ("control group"). The diagnosis of DR was established using indirect ophthalmoscopy. The rs7158663 A/G polymorphism was genotyped using real-time polymerase chain reaction (PCR) with TaqMan probes.

**Results:**

Patients with DR, compared with those without DR, had lower frequencies of both the G/G genotype (17.5% and 23.6%, respectively, p = 0.044) and the G allele (p = 0.017). When only patients with PDR were compared with controls, the G/G genotype was associated with increased protection against PDR after adjustment (odds ratio 0.551, 95% confidence interval 0.314–0.966, p = 0.038). This association also remained in the dominant (p = 0.036) and additive (p = 0.031) genetic models.

**Conclusion:**

This study reveals, for the first time, that the G/G genotype of the lncRNA *MEG3* rs7158663 single-nucleotide polymorphism is associated with a protective effect against advanced-stage DR in patients with T2DM. Additional studies are warranted to validate this finding.

## INTRODUCTION

Diabetic retinopathy (DR) is a common microvascular complication resulting from chronic effects of diabetes mellitus and a leading cause of preventable blindness and visual impairment in working-age individuals (1,2). Improvements in visual outcomes related to DR are mostly due to a combination of better systemic risk factor control and recent advances in ocular disease assessment, screening, imaging, and treatment (3).

Evidence from transcriptome analyses has demonstrated that approximately 98% of RNA molecules are not translated into proteins, representing the class of noncoding RNAs (ncRNAs) (4,5). These ncRNAs are categorized based on their length into short ncRNAs (<200 nucleotides) and long ncRNAs (>200 nucleotides), which include ribosomal RNAs and long noncoding RNAs (lncRNAs) (6). Notably, lncRNAs play a role in the progression of various pathological conditions, including the development of DR (7,8). It has been shown that changes in the genome-wide expression profile of lncRNAs accompany the development and progression of DR (7). In this context, *maternally expressed gene 3* (*MEG3*) is one of the most well-studied lncRNAs. Located on chromosome 14q32.3, *MEG3* is expressed in multiple organs. First studied as a tumor suppressor, *MEG3* plays a negative role in proliferation, apoptosis, migration, invasion, and metastasis (6,9). Moreover, *MEG3* seems to be involved in ocular diseases, as it is able to suppress neovascularization, a critical step in the development of DR (10), suggesting potential inhibitory effects of *MEG3* on DR. Conversely, *MEG3* expression is markedly downregulated in diabetic stress conditions, and its knockdown has a positive effect on retinal vascular function, including, but not limited to, endothelial cell proliferation, viability, tube formation, and anti-apoptotic action (11).

Therefore, given the biological plausibility of *MEG3* involvement in DR, we hypothesized that single-nucleotide polymorphisms (SNPs) influencing the expression of this lncRNA may be linked to susceptibility to DR. Thus, the aim of this study was to investigate the potential association between the *MEG3* rs7158663 SNP and susceptibility to DR in patients with type 2 diabetes mellitus (T2DM) from a Brazilian population.

## SUBJECTS AND METHODS

### Participants and phenotype measurements

The present case-control study was reported following the Strengthening the Reporting of Observational Studies in Epidemiology (STROBE) guidelines and Strengthening the Reporting of Genetic Association Studies (STREGA) guidelines (12,13). The study was conducted in accordance with ethical standards after the protocol was approved by the institution's ethics committee under Certificate of Presentation for Ethical Appreciation (CAAE) number 40187620.6.0000.5327.

The study enrolled 1,009 patients with T2DM. These participants were recruited from endocrinology outpatient clinics at *Hospital de Clínicas de Porto Alegre* (Rio Grande do Sul, Brazil) between January 2005 and December 2013 (14,15). The definition of T2DM followed the guidelines established by the American Diabetes Association (16). The patients were categorized according to the presence and degree of DR as follows:

- **A case group**, consisting of 628 patients with DR (345 with nonproliferative DR [NPDR] and 283 with proliferative DR [PDR]).
- **A control group**, consisting of 381 patients without DR and with diabetes duration ≥ 10 years.

An experienced ophthalmologist assessed patients for DR using indirect ophthalmoscopy on dilated pupils. Patients in the control group (*i.e.*, those without DR) exhibited no abnormalities in the retina. Patients in the NPDR group presented microaneurysms, hemorrhage, and hard exudates, while those in the PDR group presented newly formed blood vessels and/or growth of fibrous tissue into the vitreous cavity (17). The DR classification was determined by the most severe degree of retinopathy in the worst affected eye (1), according to the Global Diabetic Retinopathy Group scale (17).

A standard questionnaire was utilized to collect information about the patients’ age, age at diabetes diagnosis, and drug treatment. Ethnicity was defined based on self-classification. The laboratory assessment of these patients has been detailed in previous publications (14,18). Body mass index (BMI) was calculated as weight (in kg) divided by height squared (in meters squared) ². Hypertension was defined as blood pressure levels ≥ 140/90 mmHg or the use of antihypertensive medication.

### Genotyping

For genotyping, DNA was extracted from peripheral blood leucocytes using the FlexiGene DNA kit (QIAGEN, Germantown, MD, USA). The *MEG3* rs7158663 A/G SNP was genotyped by real-time polymerase chain reaction (PCR) using TaqMan MGB probes included in the Human TaqMan Genotyping Assay 40x, ID = C_9693465_10 (Thermo Fisher Scientific, Foster City, CA, USA). All reactions were conducted in 384-well plates, in a final volume of 5 μL, containing 1 μL of DNA, 0.25 μL of TaqMan Genotyping Assay 20x, and 2.50 μL TaqPath ProAmp 1 X Mastermix (Thermo Fischer Scientific). The plates were run in a real-time PCR thermal cycler (ViiA7 Real-Time PCR System; Thermo Fisher Scientific) and heated for 10 min at 95 °C, followed by 50 cycles of 95 °C for 15 seconds and 62 °C for 1 minute. The amplification reactions were performed twice in 10% of the samples. The genotyping success rate exceeded 95%, with a calculated error rate based on PCR duplicates of 0.5%.

### Statistical analysis

Allele frequencies were determined by direct counting, and deviations from the Hardy-Weinberg equilibrium (HWE) were assessed using the chi-square test. Genotypes were also compared between groups under additive, recessive, and dominant inheritance models, following the categories suggested by Zintzaras and Lau (19). Clinical and laboratory characteristics between groups were compared using analysis of variance (ANOVA), Student's *t* test, or chi-square test, as appropriate. Categorical variables are shown as frequency (%). Quantitative variables with normal distribution are shown as mean ± standard deviation (SD), while quantitative variables with skewed distribution (which were analyzed after log-transformation) are shown as median (25th-75th percentile values). The Kolmogorov-Smirnov and Shapiro-Wilk tests were used to assess the normality of these variables. Logistic regression analyses were performed to analyze the association of the SNP of interest with DR or PDR, adjusting for confounding variables. Those variables associated with DR or PDR in the univariate analyses were included in the multivariate model.

Sample sizes were calculated using the OpenEpi software (available at www.openepi.com). Thus, using the frequencies from a previous study that evaluated the association of the *MEG3* rs7158663 A/G SNP with T2DM (20) (minor allele frequency = 0.20), the calculated sample size was 577 individuals for the case group and 231 individuals for the control group, to detect an odds ratio (OR) of 1.7 with 80% power and an alpha level of 0.05.

The statistical analyses were performed using SPSS 18.0 for Windows (IBM Corp., Armonk, NY, USA).

## RESULTS

### Clinical features of the patients categorized by the presence or absence of diabetic retinopathy

The main clinical and laboratory characteristics of the patients, stratified according to DR presence (case group; patients with T2DM and DR) or absence (control group; patients with T2DM and without DR) are presented in Table 1. Compared with the case group, the control group had a higher prevalence of female patients (46.5% and 62.6%, respectively, p < 0.001) and individuals self-identified as White (75.4% and 81.4%, respectively, p = 0.036). The control group, compared with the case group, also had a higher mean age (p < 0.001), BMI value (p = 0.002), and lower glycated hemoglobin (HbA1c) level (p = 0.007). As expected, the presence of hypertension and diabetic kidney disease (DKD) was more frequent in the case group compared with the control group (p = 0.002 and p < 0.001, respectively).

**Table 1:** Clinical and laboratory characteristics of patients with type 2 diabetes mellitus categorized by the presence (case group) or absence (control group) of diabetic retinopathy

Characteristic	Control group (n = 381)	Case group (n = 628)	P values*
Sex (% females)	238 (62.6)	292 (46.5)	<0.001
Ethnicity (% white)	298 (81.4)	464 (75.4)	0.036
Hypertension (%)	295 (84.5)	518 (91.4)	0.002
Age (years)	69.3 ± 10.6	65.7 ± 10.4	<0.001
BMI (kg/m²)	29.1 ± 5.3	28.0 ± 5.1	0.002
Total cholesterol (mg/dL)	194.1 ± 51.1	195.2 ± 52.0	0.748
HDL cholesterol (mg/dL)	46.6 ± 12.6	44.8 ± 13.9	0.044
LDL cholesterol (mg/dL)	111.6 ± 45.4	115.6 ± 46.6	0.220
Triglycerides (mg/dL)	152.0 (106.0-220.0)	151.0 (103.0-219.0)	0.850
HbA1c (%)	7.3 ± 1.8	7.8 ± 2.1	0.007
DKD (%)	187 (49.1)	484 (77.1)	<0.001

Data are shown as mean ± standard deviation, median (25th-75th percentiles) or frequency (%).

* P values were computed using Student's *t* test or chi-square test, as appropriate. The control group comprised patients with type 2 diabetes mellitus for over 10 years and without diabetic retinopathy.

Abbreviations: BMI, body mass index; DKD, diabetic kidney disease; DR, diabetic retinopathy; HbA1c, glycated hemoglobin; HDL cholesterol, high-density lipoprotein cholesterol; LDL cholesterol, low-density lipoprotein cholesterol.

### Genotype and allele frequencies in patients categorized by the presence or absence of diabetic retinopathy

The genotype and allele frequencies of the *MEG3* rs7158663 A/G SNP in patients with DR (case group, encompassing patients with PDR and those with NPDR) and patients without DR (control group) are presented in Table 2. Of note, the genotype frequencies of this SNP were in agreement with those predicted by the HWE in the case and control groups (p > 0.05). The G allele had a frequency of 41.8% in patients with DR and 47.2% in those without DR (p = 0.017). The frequency of the G/G genotype was also lower in patients with *versus* without DR (17.5% *versus* 23.6%, p = 0.044). The lower frequency of the G/G genotype in patients with DR, compared with those without DR, was also observed in the additive and recessive genetic models (p = 0.017 and p = 0.023, respectively). After adjustment for covariates (sex, age, BMI, and presence of hypertension and DKD), the G/G genotype remained associated with protection against DR (OR = 0.660, 95% confidence interval [CI] 0.437–0.998, p = 0.049). The association also remained in the additive model adjusted for the same covariates (OR = 0.657, 95% CI 0.434-0.996, p = 0.048).

**Table 2:** Genotype and allele distributions of *MEG3* rs7158663 A/G single-nucleotide polymorphisms in patients with type 2 diabetes mellitus categorized according to the presence (case group) or absence (control group) of diabetic retinopathy

rs7158663	Control group (n = 381)	Case group (n = 628)	P*	Adjusted OR (95% CI) /† P
Genotype				
A/A	111 (29.2)	214 (34.1)	0.044	1
A/G	180 (47.2)	304 (48.4)		0.776 (0.553-1.088)/0.142
G/G	90 (23.6)	110 (17.5)		0.660 (0.437-0.998)/0.049
A	0.528	0.582	0.017	-
G	0.472	0.418		
**Dominant model**				
A/A	111 (29.1)	214 (34.1)	0.119	1
A/G+G/G	270 (70.9)	414 (65.9)		0.739 (0.538-1.015)/0.062
**Recessive model**				
A/A+A/G	291 (76.4)	518 (82.5)	0.023	1
G/G	90 (23.6)	110 (17.5)		0.770 (0.538-1.101)/0.152
**Additive model**				
A/A	111 (55.2)	214 (66.0)	0.017	1
G/G	90 (44.8)	110 (34.0)		0.657 (0.434-0.996)/0.048

Data are shown as frequency (%) or proportion.

* P value*s* were calculated using the chi-square test.

† P values and odds ratios (95% confidence intervals) were calculated using logistic regression analysis adjusted for sex, age, body mass index, and presence of hypertension and diabetic kidney disease. The control group comprised patients with type 2 diabetes mellitus for over 10 years and without diabetic retinopathy.

Abbreviations: 95% CI, 95% confidence interval; DR, diabetic retinopath; y; T2DM, type 2 diabetes mellitus.

### Genotype and allele frequencies in patients with proliferative diabetic retinopathy compared with controls

When we further categorized the patients with DR (case group) according to DR severity (*i.e.*, PDR *versus* NPDR), we observed that the protective effect of the G allele was linked to DR severity (G/G frequency: control group, 23.6%; NPDR group, 19.9%; PDR group, 14.8%; p = 0.043 and p for trend = 0.003). Since the difference in G/G frequency was particularly notable between patients with PDR *versus* controls, we excluded the patients with NPDR from subsequent analyses and focused on comparing only those with PDR *versus* controls.

As shown in Table 3, the G/G genotype conferred protection against PDR ((p = 0.008). This protective association persisted across the dominant (p = 0.037), additive (p = 0.002), and recessive (p = 0.007) models. After adjustment for age, sex, BMI, ethnicity, and presence of hypertension and DKD, the G/G genotype remained associated with protection against PDR (OR = 0.551, 95% CI 0.314-0.966, p = 0.038). This association also remained in the dominant (OR = 0.641, 95% CI 0.423-0.972, p = 0.036) and additive (OR = 0.540, 95% CI 0.308-0.947, p = 0.031) models.

**Table 3:** Genotype and allele distributions of *MEG3* rs7158663 A/G single-nucleotide polymorphisms in patients with type 2 diabetes mellitus categorized according to the presence of proliferative diabetic retinopathy *versus* absence of diabetic retinopathy (controls)

rs7158663	Controls (n = 381)	Patients with PDR (n = 283)	P*	Adjusted OR (95% CI) /† P
**Genotype**				
A/A	111 (29.2)	105 (37.1)	0.008	1
A/G	180 (47.2)	136 (48.1)		0.681 (0.438-1.058)/0.087
G/G	90 (23.6)	42 (14.8)		0.551 (0.314-0.966)/0.038
A	0.528	0.611	0.002	
G	0.472	0.389		
**Dominant model**				
A/A	111 (29.1)	105 (37.1)	0.037	1
A/G+G/G	270 (70.9)	178 (62.9)		0.641 (0.423-0.972)/0.036
**Recessive model**				
A/A+A/G	291 (76.4)	241 (85.2)	0.007	1
G/G	90 (23.6)	42 (14.8)		0.696 (0.425-1.140)/0.150
**Additive model**				
A/A	111 (55.2)	105 (71.4)	0.002	1
G/G	90 (44.8)	42 (28.6)		0.540 (0.308-0.947)/0.031

Data are shown as frequency (%) or proportion.

* P values were calculated using the chi-square test.

† P values and odds ratios (95% confidence intervals) were calculated using logistic regression analysis adjusted for sex, age, body mass index, ethnicity, and presence of hypertension and diabetic kidney disease. The control group comprised patients with type 2 diabetes mellitus for over 10 years and without diabetic retinopathy.

Abbreviations: 95% CI, 95% confidence interval; T2DM, type 2 diabetes mellitus.

## DISCUSSION

Findings from current research suggest that specific genetic variations within lncRNA genes may be linked to the development of diseases (21-23). However, limited research has explored the connection between SNPs within lncRNAs and DR. In the present study, we evaluated the frequency of the lncRNA *MEG3* rs7158663A/G SNP in patients with T2DM categorized according to the presence and severity of DR. The results of our study demonstrate for the first time that the G/G genotype of the *MEG3* rs7158663A/G SNP is associated with a protective effect against PDR in patients with T2DM.

Recent evidence indicates that epigenetic factors contribute to the initiation and severity of DR. MicroRNAs (miRNAs), a class of short ncRNAs, have emerged as critical regulators of gene expression and key players in the complex molecular mechanisms underlying DR development (24). In contrast to miRNAs, which exert their negative regulatory influence by binding to the 3’ untranslated region (UTR) of their target genes, lncRNAs exhibit a more diverse range of functions. Indeed, lncRNAs play crucial regulatory roles in cellular processes, including the modulation of gene expression, chromatin organization, and regulation of various signaling pathways (25). They also interact with miRNAs, acting as molecular sponges to control the concentration of cytoplasmic miRNAs. This negative regulation of miRNAs, in turn, leads to the upregulation of their target mRNAs (26).

In the context of diabetes, *MEG3* expression has been described as being notably downregulated in islets from patients with T2DM compared with those from controls without diabetes (27). Furthermore, You and cols. (28) showed that the inhibition of *MEG3* expression leads to reduced insulin secretion and impaired glucose tolerance. The lncRNA *MEG3* has also been implicated not only in insulin resistance but also in ocular diseases (11,29). Downregulation of *MEG3* expression has been observed in the retinas of diabetic mice and in retinal endothelial cells subjected to elevated glucose-induced stress (11). Additionally, *MEG3* overexpression results in reduced miR-34a levels and increased SIRT1 levels in retinal cells, thereby inhibiting hyperglycemia-induced apoptosis and secretion of inflammatory cytokines (29). The *MEG3* effect was attributed to the inhibition of the NF-κB signaling pathway and increased Bcl-2/Bax ratio via downregulation of *miR-34a* (29). Similarly, *MEG3* upregulation effectively suppresses the development of retinal neovascularization in mice with oxygen-induced retinopathy through downregulation of PI3K, serine/threonine kinase, vascular endothelial growth factor (VEGF), and proinflammatory factors (30). Moreover, *MEG3* knockdown has a harmful impact on retinal vascular function, resulting in severe capillary degeneration, increased microvascular leakage, and inflammation (11). The *MEG3* knockdown also influences negatively the proliferation, migration, and tube formation of retinal endothelial cells (11). The primary mechanism by which *MEG3* influences endothelial cell function seems to be via the activation of the phosphoinositide 3-kinase (PI3K)/protein kinase B (Akt) signaling pathway (11). These studies suggest that *MEG3* primarily inhibits the excessive proliferation of retinal endothelial cells in DR. Therefore, increasing *MEG3* expression may hold promise for protecting the retina from pathological neovascularization and inflammation.

A bioinformatics analysis has shown that the *MEG3* rs7158663 SNP has the potential to alter the *MEG3* RNA folding structure and impact miRNA-*MEG3* interactions, subsequently influencing the expression of their target miRNAs and/or expression of *MEG3* (31). Several studies have explored the association of this potentially functional SNP as a genetic marker for predicting the risk of various cancers; however, additional validation is required. Notably, a meta-analysis has reported that the *MEG3* rs7158663 A/A genotype is associated with an increased risk of gastric and colorectal cancers (30). Interestingly, it has also been shown that the *MEG3* rs7158663 A/A genotype is linked to T2DM risk (20). The *MEG3* rs7158663 G/G genotype, compared with the A genotype, has been associated with lower creatinine levels and higher estimated glomerular filtration rates in patients with T2DM (18). This last result aligns with the findings of the present study, which showed that the G/G genotype conferred protection against PDR. Although PDR and DKD share some common underlying mechanisms, this association persisted after correcting for the presence of DKD. Therefore, we hypothesize that the G/G genotype may influence the regulation of *MEG3* expression and, thus, affect angiogenesis, inflammation, endothelial cell function, and vascular smooth muscle cell proliferation pathways within the context of vascular diseases, such as PDR (6,10,32).

Despite the strengths of the present study, it has some limitations that must be acknowledged. The protocol for diagnosis of DR relied on a clinical examination and did not include retinal photography, which is the reference method for diagnosis of DR, or other methods with better performance, such as angiography or optical coherence tomography (OCT). Additionally, the clinical evaluation of DR was conducted by a single examiner. Although this examiner was an experienced ophthalmologist, the assessment may still be subject to bias. Furthermore, since this genetic study included a limited sample size, the results might only be applicable to the specific group studied.

In summary, our results provide initial evidence suggesting an association between the *MEG3* rs7158663 G/G genotype and protection against PDR in patients with T2DM. Further research involving diverse ethnic groups, as well as functional studies, are required to further elucidate the relationship between this SNP and DR.
